# A MultiSite Gateway^TM ^vector set for the functional analysis of genes in the model *Saccharomyces cerevisiae*

**DOI:** 10.1186/1471-2199-13-30

**Published:** 2012-09-20

**Authors:** Astrid Nagels Durand, Tessa Moses, Rebecca De Clercq, Alain Goossens, Laurens Pauwels

**Affiliations:** 1 Department of Plant Systems Biology, VIB, Technologiepark 927, B-9052, Gent, Belgium; 2 Department of Plant Biotechnology and Bioinformatics, Ghent University, Technologiepark 927, B-9052, Ghent, Belgium; 3 Laboratory of Molecular Cell Biology, Institute of Botany and Microbiology, Katholieke Universiteit Leuven, Kasteelpark Arenberg 31, B-3001, Leuven-Heverlee, Belgium; 4 Department of Molecular Microbiology, VIB, Kasteelpark Arenberg 31, B-3001, Leuven-Heverlee, Belgium

**Keywords:** Gateway cloning, MultiSite, *Saccharomyces cerevisiae*, Yeast, Vector, Fusion protein, Epitope tag, Jasmonate, *Arabidopsis thaliana*

## Abstract

**Background:**

Recombinatorial cloning using the Gateway^TM^ technology has been the method of choice for high-throughput omics projects, resulting in the availability of entire ORFeomes in Gateway^TM^ compatible vectors. The MultiSite Gateway^TM^ system allows combining multiple genetic fragments such as promoter, ORF and epitope tag in one single reaction. To date, this technology has not been accessible in the yeast *Saccharomyces cerevisiae*, one of the most widely used experimental systems in molecular biology, due to the lack of appropriate destination vectors.

**Results:**

Here, we present a set of three-fragment MultiSite Gateway^TM^ destination vectors that have been developed for gene expression in *S. cerevisiae* and that allow the assembly of any promoter, open reading frame, epitope tag arrangement in combination with any of four auxotrophic markers and three distinct replication mechanisms. As an example of its applicability, we used yeast three-hybrid to provide evidence for the assembly of a ternary complex of plant proteins involved in jasmonate signalling and consisting of the JAZ, NINJA and TOPLESS proteins.

**Conclusion:**

Our vectors make MultiSite Gateway^TM^ cloning accessible in *S. cerevisiae* and implement a fast and versatile cloning method for the high-throughput functional analysis of (heterologous) proteins in one of the most widely used model organisms for molecular biology research.

## Background

The model organism *Saccharomyces cerevisiae* has contributed greatly to our current understanding on eukaryotic genes, their products, and their functions. Decades of study have resulted in an extensive knowledge on yeast physiology, genetics, and the molecular functions and interactions of its proteins. Furthermore, this unicellular eukaryotic system is well suited for the study of basic cellular processes which are often conserved in higher eukaryotes. Because of its ease for genetic modification and fast growth, yeast became the system of choice for *in vivo* protein analyses from other eukaryotes. *S. cerevisiae* was used, for example, to perform proteome-wide analysis of the human protein-protein interaction networks [1], to systematically analyze protein-DNA interaction networks of the nematode *C. elegans*[2], and to produce high-value bioactive plant secondary metabolites through metabolic engineering approaches [3].

**Figure 1:**
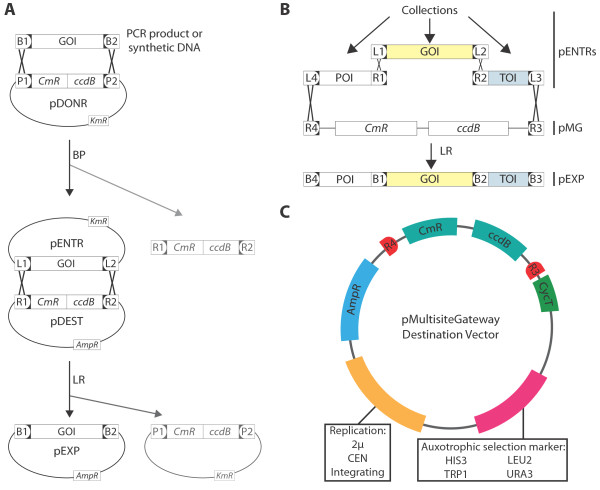
**Schematic overview of (MultiSite) Gateway**^**TM **^**cloning and properties of the constructed destination vectors. **(**A**) Overview of the Gateway^TM ^cloning procedure. Attachment of *attB *sites to a DNA segment of choice, often generated by PCR or as synthetic DNA, allows recognition by the BP Clonase^TM ^and recombination into a donor vector (pDONR) containing *attP *sites, yielding an entry clone (pENTR) carrying *attL *sites. The DNA segment in the pENTR clone can then be transferred to a destination vector (pDEST) by recombination between *attL *and *attR *sites present on the pDEST vector mediated by the LR Clonase^TM^. This yields an expression clone (pEXP) in which the DNA segment again becomes flanked by *attB *sites. Positive selection of pENTR and pEXP clones on medium containing appropriate antibiotics together with negative selection of starting products and by-products (shown in grey) based on the presence of a negative (*ccdB*) selection marker between the recombination sites (Gateway^TM ^Cassette) further increases the efficiency of the system. (**B**) A combination of existing pENTRs, or new pENTRs, are easily assembled in a single MultiSite Gateway^TM ^reaction catalyzed by the LR II Clonase^TM ^Plus using pMG as destination vector. (**C**) Schematic representation of the MultiSite Gateway^TM ^compatible vector set (pMG) for transformation of *S. cerevisiae*. GOI, POI and TOI indicate gene, promoter and tag of interest, respectively. CmR, AmpR and KmR indicate chloramphenicol, ampicillin and kanamycin resistance, respectively. B1-B4, P1-P2, L1-L4 and R1-R4 stand for the respective *att *sites. CycT indicates the CYC1 terminator on the vector’s backbone.

**Figure 2:**
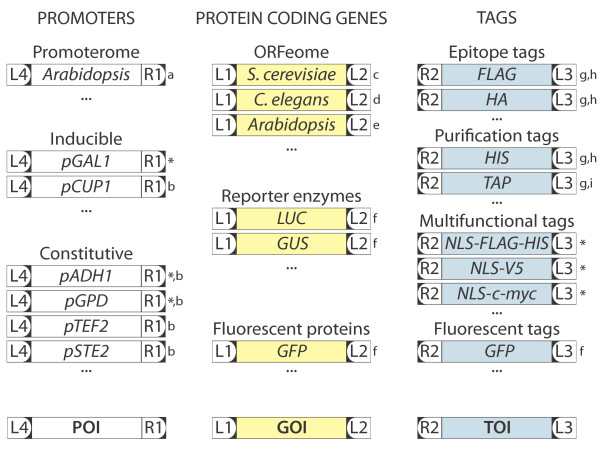
**Flexibility of the MultiSite Gateway™ cloning system. **The flexibility of the MultiSite Gateway^TM ^system is illustrated by the ample possibilities to combine any gene of interest (GOI) with any promoter of interest (POI) and tag of interest (TOI), depending on the experimental needs. Many of such sequences, including whole ORFeomes (cloned without STOP codon) of several model organisms [16-19] and a broad range of tags [5,9-11] are already available in suitable pDONR vectors. L1-L4 and R1-R2 stand for the *att* sites *attL1attL4 *and *attR1attR2*, respectively. Asterisks mark constructs generated in this study, lower case letters indicate constructs generated previously by others: a [8], b [30], c [19], d [16], e [5], f [9], g [10],h [11].

Large scale genomics approaches to uncover protein function are adopted more and more in the current era of systems biology research. To cope with the large amounts of constructs needed, scientists make use of high-throughput cloning technologies, such as the Gateway^TM^ technology (Invitrogen; http://www.invitrogen.com/), which is based on the site-specific recombination system from the bacteriophage lambda that facilitates the integration of the phage’s DNA into the *Escherichia coli* chromosome [4]. DNA segments that are flanked by the appropriate recombination sites in a standard vector (pENTR) can easily be transferred to a compatible vector (pDEST) for functional analysis (Figure 1A). MultiSite Gateway^TM^ uses modified recombination sites to allow the combination of multiple DNA segments in one single *in vitro* recombination reaction. The segments are joined in a pDEST in a predefined order and orientation, maintaining the reading frame and with low risk for mutations [4] (Figure 1B). Three-segment MultiSite Gateway^TM^ makes it possible to easily make any combination of a promoter, gene and tag without the need of redesigning new destination vectors for each new experimental approach. The ability to choose a promoter allows varying temporal, spatial, and quantitative control of gene expression, while different possible tags enable the inclusion of fluorescent protein tags for localisation or epitope tags for detection or purification, or the creation of protein chimeras (Figure 2). The flexibility introduced by the MultiSite Gateway^TM^ technology is illustrated by the large amount of “building blocks” already made available as pENTR clones by several research groups [5-11]. Existing pENTR collections include ORFeomes from multiple prokaryotic and eukaryotic organisms [12-26].

Single-segment pDEST vectors are available for virtually all commonly used systems, such as Drosophila [27]. Moreover, for plants an extensive repertoire of pDEST for two- and three-segment MultiSite Gateway^TM^ has been established [28]. Three-segment MultiSite Gateway^TM^ destination vectors are also available for Gram-positive bacteria [29]. In *S. cerevisiae*, single-segment pDEST vectors are available for yeast two-hybrid screens (Invitrogen), and two-segment (promoter::ORF) MultiSite Gateway^TM^ vectors have been described [30]. An extensive set of single-fragment Gateway^TM^ vectors was constructed, allowing N-terminal fusions with four fluorescent tags, and C-terminal fusions with five different fluorescent tags, an affinity tag and an epitope tag under the control of the inducible GAL1 or constitutive GPD promoter [31]. Alternatively, tags that are not present in this vector set can be fused to the gene of interest through 2-step PCR fusion [32] before performing the BP reaction. However, to our knowledge no three-segment MultiSite Gateway^TM^ pDEST vectors exist for *S. cerevisiae* to date. As a consequence, a large number of commonly used protein tags already available as pENTR clones are not readily applicable in the organism that - together with *E. coli* - is the workhorse of molecular biology.

In this paper we present a set of eleven functionally validated three-segment MultiSite Gateway^TM^ pDEST vectors for use in *S. cerevisiae*. The vector set features the four most commonly used auxotrophic markers for selection of yeast transformants combined with three different replication mechanisms. The presence of the CYC1 terminator in these vectors allows construction of any promoter::ORF:tag combination. In addition, a number of useful entry clones harbouring commonly used yeast promoters and entry clones with protein tags to be used with these pDESTs are presented and validated. These vectors have been appended to our collection of ‘Gateway™ vectors for functional studies’ and can be ordered through the website http://gateway.psb.ugent.be/. Finally, we illustrate the applicability of this vector set by confirming the formation of a ternary complex between the jasmonate ZIM-domain (JAZ), the Novel Interactor of JAZ (NINJA) and the TOPLESS (TPL) proteins, with NINJA acting as a bridging protein. This complex was previously shown to be involved in jasmonate signalling in the model plant Arabidopsis (*Arabidopsis thaliana*) [33].

## Results and discussion

### Construction of MultiSite Gateway^TM^ vectors

The MultiSite Gateway^TM^ cassette of pKm43GW (http://gateway.psb.ugent.be/) flanked by *attB4* and *attB3* sites, was PCR amplified adding *Xho*I and *Sac*I restriction sites (Additional file 1). The amplification product was subsequently cloned into the *Xho*I and *Sac*I sites present in the backbones of the vectors of the pAG-series [31]. This step replaced the original Gateway^TM^ cassette and the eukaryotic promoter from the pAG vectors, while maintaining the CYC1 terminator. Ligation products were transformed into *ccdB* resistant *E. coli* cells (One shot *ccdB* survival^TM^, Invitrogen). The pAG vectors contain an ampicillin resistance gene for selection in *E. coli*, one of four different auxotrophic selection markers *(HIS3, LEU2, TRP1*, and *URA3*) and one of three different replication determinants (2 μ ori, CEN, or Integrating) thus giving rise to a MultiSite Gateway^TM^ vector set (Figure 1C) for expression in *S. cerevisiae* (see Table 1 for nomenclature). We did not succeed in creating the Integrating vector with the *HIS3* autotrophic marker (Table 1).

**Table 1:** **Nomenclature of the *S. cerevisiae *MultiSite Gateway**^**TM **^**pDEST vectors**

Name*	**Replication**	**Selectable marker**
pMG304	Integrating	TRP1
pMG305	Integrating	LEU2
pMG306	Integrating	URA3
pMG413	CEN	HIS3
pMG414	CEN	TRP1
pMG415	CEN	LEU2
pMG416	CEN	URA3
pMG423	2 μ	HIS3
pMG424	2 μ	TRP1
pMG425	2 μ	LEU2
pMG426	2 μ	URA3

*pMG*XYZ *where MG indicates the presence of the MultiSite Gateway^TM ^cassette; *X *stands for the presence (4) or absence (3) of replication determinants on the vector; *Y* stands for the replication determinant: Integrating (0), CEN (1) or 2 μ (2); *Z* indicates the auxotrophic selection marker: HIS3 (3), TRP1 (4), LEU2 (5) or URA3 (6).

### Testing the versatility of the system

**Figure 3:**
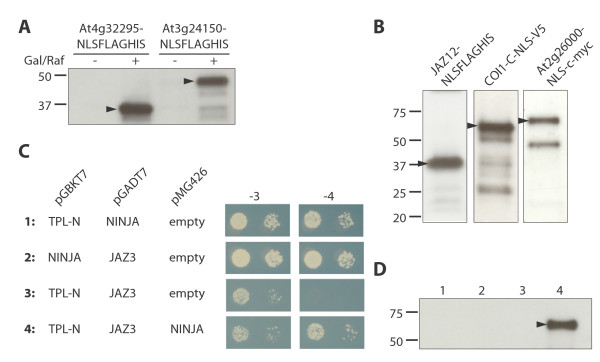
**Versatility of the newly generated MultiSite Gateway**^**TM **^**vector set. ** (**A**) Inducibility of the pGAL1 promoter. Arabidopsis genes At4g32295 and At3g24150 were expressed in yeast under the control of the pGAL1 promoter and fused with an NLS-FLAG-HIS tag using the pMG416 vector. After total protein extraction, expression was verified in inducing (+) and non-inducing (−) medium through immunoblot analysis with anti-FLAG antibodies. (**B**) Functionality of newly developed C-terminal translational fusion epitope tags. Yeasts were transformed with pMG425-ADH1::JAZ12:−NLS-FLAG-HIS, pMG423-GPD::COI1-C:−NLS-V5 where COI1-C is a truncated version of COI1, or pMG423-GPD::At2g26000: NLS-c-myc. Expression of the different constructs was verified with immunoblot analysis of total protein extracts with anti-FLAG, anti-V5 and anti-c-myc antibodies, respectively. (**C**) Interaction of proteins in the trimeric JAZ3-TPL-NINJA complex in Y3H assays. Transformed yeasts were spotted in 10-fold and 100-fold dilutions on control medium (−3: SD-Leu-Trp-Ura) and selective medium (−4: SD-His-Leu-Trp-Ura). Gene constructs in the pGBKT7 and pGADT7 vectors carry the DNA-binding domain or the transcription activation domain, respectively, in contrast to constructs expressed in pMG426, which do not carry DNA-binding or transcription activation domains. (**D**) Verification of expression of NINJA: NLS-FLAG-HIS in the Y3H set-ups shown in panel C. After total protein extraction, NINJA: NLS-FLAG-HIS was detected through immunoblot analysis with anti-FLAG antibodies. Arrowheads indicate the fusion protein.

To illustrate the flexibility of this vector collection, we took advantage of a few “building blocks” for MultiSite Gateway^TM^ cloning that were available in-house as entry clones (Figure 2, http://gateway.psb.ugent.be/). One of the assets of MultiSite Gateway^TM^ destination vectors is that any promoter of interest can be used, provided it is available as an entry clone flanked by *attL4* and *attR1* sites. To achieve this, we cloned two constitutively active promoters (pGPD/TDH3 and pADH1) and a galactose-inducible promoter (pGAL1) into pDONR P4-P1R (Invitrogen). The inducibility of the GAL1 promoter was verified by expression in yeast of a heterologous Arabidopsis gene fused to a NLS-FLAG-HIS tag under the control of the GAL1 promoter using pMG416 (Figure 3A).

Another advantage of the MultiSite Gateway^TM^ system is that several different tag sequences are readily available (Figure 2) and can easily be introduced to acquire translational fusions, since the *att* sites do not disturb the reading frame. For proof-of-concept, we cloned different plant genes (without STOP codon) in a C-terminal translational fusion with either a V5, c-myc, or FLAG-HIS tag. To make these constructs compatible with protein interaction studies in yeast, we additionally fused each epitope tag with a nuclear localization signal (NLS) derived from SV40, thereby creating the NLS-3xV5, NLS-3xc-myc, and NLS-3xFLAG-6xHIS tags, respectively. Such constructs allow avoiding false negative experimental outcomes that result from protein localisation in different cellular compartments, for instance. Tagged plant proteins were expressed in yeast under the control of the constitutive GPD or ADH1 promoters in different vectors from our set. Total protein extracts were obtained and the expressed proteins were visualized through immunoblot analysis (Figure 3B). The (NLS)FLAG-HIS tag is particularly suitable when protein purification under denaturing conditions is needed, while, at the same time, low background detection on immunoblots is desired.

To express C-terminal translational fusions by means of our promoters, tags and destination vectors, entry vectors containing the GOI should be cloned with a start codon and without a stop codon, and the manufacturer’s guidelines regarding the reading frame should be adopted (http://www.invitrogen.com). Organisms for which ORFeome collections contain vectors that match these criteria include several bacterial collections (*Brucella melitensis*[12], *Pseudomonas aeruginosa*[13], *Bacillus anthracis*, *Francisella tularensis*, *Helicobacter pylori*, *Mycobacterium tuberculosis*, *Rickettsia prowazekii*, *Staphylococcus aureus*, *Streptococcus pneumoniae*, *Vibrio cholerae*[14]), human and mouse [15], plants (Arabidopsis [16], maize, sorghum, sugarcane, rice [17]), viruses [18] and yeasts (*S. cerevisiae*[19] and *Schizosaccharomyces pombe*[20]). Caution is advised since some ORFeome collections contain entry clones both with and without stop codon [15,16]. ORFeome collections that comprise only GOI’s provided with a stop codon, as is the case for some bacterial and viral ORFeomes (*Neisseria gonorrhoeae*[21], *Sinorhizobium meliloti*[22], *Yersinia pestis*[14], and Hepatitis C [23]) are not compatible with our vectors. Other ORFeomes in which the GOI is cloned without a start and without a stop codon (*Caenorhabditis elegans*[24,25], *E. coli*[26]), are not compatible with our current promoters but can be used for expression of C-terminal translational fusions with our vectors provided the promoter is cloned followed by a start codon. Since this ORFeome list is non-exhaustive and, as described above, different approaches are used when cloning the GOI, the compatibility of available pENTR collections should always be corroborated either *in silico* before assembling the different sequences into expression vectors or through epitope tag detection in immunoblots. Finally, the destination vector set presented here also allows expression of N-terminal fusions, provided the *att* sites flanking the different fusion components are adapted accordingly.

### Value of the vector set

The value of this vector set was exemplified by a yeast three-hybrid (Y3H) experiment, in which we investigated the formation of the ternary protein complex by the Arabidopsis JAZ3, NINJA, and TPL proteins. We have proposed recently that the adaptor protein NINJA bridges the JAZ proteins to TPL proteins and thereby forms a repressor complex that blocks the cellular programs regulated by the jasmonates, ubiquitous plant hormones that regulate various aspects of plant growth, development, and survival [33]. TPL interacts with the ETHYLENE RESPONSIVE FACTOR–associated amphiphilic repression (EAR) motif that is present in NINJA [33] but absent in most JAZ proteins, including JAZ3. Some JAZ proteins however, i.e. JAZ5 to JAZ8, contain EAR motifs themselves and are capable of direct interaction with TPL [34-36].

The N-terminal domain of TPL contains the LisE and CTHL domains and was previously shown to be essential for binding the EAR motif in Aux/IAA proteins [37]. Therefore we cloned this part (denominated TPL-N) as a bait protein for Y2H. In agreement with the proposed models, the interaction of TPL-N with NINJA was confirmed but TPL-N could not interact with JAZ3 (Figure 3C).

Commonly used Y2H vectors such as pGADT7 and pGBKT7 (Clontech) are designed such that the bait and prey fusion proteins are targeted to the same subcellular compartment (i.e. nucleus) and are equipped with an epitope tag, HA and c-myc, respectively, allowing easy confirmation of expression through immunoblot. To verify whether NINJA can connect EAR-lacking JAZs with TPL, as previously proposed [33], we performed a Y3H assay in which we expressed NINJA, under control of a constitutive promoter (pGPD), as a bridging protein. Hereby we used the MultiSite Gateway^TM^ vector pMG426 (Table 1) that carries the URA3 auxotrophic marker that is often still available in yeast strains used for Y2H. As a C-terminal tag we used the NLS-FLAG-HIS tag. Only when NINJA is co-expressed, yeast growth was observed on selective –His medium, indicating that interaction between JAZ3 and TPL requires the involvement of NINJA (Figure 3C-D).

## Conclusions

We have successfully constructed a set of three-segment MultiSite Gateway^TM^ destination vectors for *S. cerevisiae*. Our findings make high-throughput recombinatorial cloning of multiple genetic segments in one single reaction accessible in one of the most widely used experimental model systems in molecular biology. The availability of different auxotrophic markers in this vector set, together with the large amount of existing compatible building blocks for MultiSite Gateway^TM^ cloning already available in several research groups, creates a versatile utility for these vectors. In addition, we have cloned two constitutive and one inducible yeast promoter in appropriate pENTR vectors and constructed three novel epitope tags, each including a NLS, which are suitable for interaction studies in yeast.

The usefulness of the MultiSite Gateway^TM^ vectors was demonstrated in a Y3H assay with which we corroborated the hypothesis that NINJA connects the JAZ proteins with the co-repressor TPL. This trimeric complex mediates repression of jasmonate responsive genes in the absence of the hormone [33].

Implementation of the vector set presented in this article, together with the cloning of more promoters and (epitope) tags according to personal experimental needs, will facilitate gene functional studies and contribute to the high-throughput versatile expression of heterologous (plant) proteins in yeast.

## Methods

### Strains and growth conditions

The *E. coli* strains used were either the *ccdB* resistant strain DB3.1 (Invitrogen) or the *ccdB* sensitive strain DH5α. Both were grown at 37°C in LB broth medium with appropriate antibiotics. Several different commonly used yeast lab-strains were grown at 30°C in synthetic defined medium (Clontech) lacking the appropriate amino acids.

### MultiSite Gateway^TM^ cloning and yeast transformation

MultiSite LR reactions were performed in 10 μL total volume containing 10 fmoles of each entry vector, 20 fmoles of destination vector, and 2 μL LR II Clonase^TM^ Plus (Invitrogen). The reaction was incubated overnight at 25°C. After proteinase treatment, the mix was transformed into *E. coli* DH5α. Colonies that grew on selective medium were picked and the insert was sequenced using M13 forward and reverse primers (Additional file 1). To maintain the reading frame, necessary for expression of translational fusions, MultiSite Gateway^TM^ cloning was carried out according to the manufacturer’s guidelines (http://www.invitrogen.com). Note that in order to produce C-terminal translational fusions, the ORFs used should be without STOP codon. A convenient method to obtain simultaneously clones of ORFs with and without STOP codon has been described [38].

Competent yeast cells were transformed using the LiAc/SS carrier DNA/PEG method [39].

### Promoter cloning

The *attB4* and *attB1* sites were introduced in the primers used for promoter amplification (Additional file 1). A PCR was performed using Phusion High-Fidelity PCR Kit (Thermo Fisher Scientific) on 50 ng of pBEVY-A, pBEVY-GL [40], and pGAD424 (Clontech) as template for the GPD, GAL, and ADH1 promoters, respectively. PCR products were purified with the GeneJET Gel Extraction kit (Fermentas). BP reactions were performed in a total volume of 5 μl containing 1 μl enzyme, 300 ng pDONR P4-P1R (Invitrogen), and 30 ng of PCR product. Incubation and subsequent treatments were the same as those for MultiSite LR reactions.

### Epitope-tag design and immunoblot analysis

Synthetic DNA encoding NLS-3xFLAG-6xHIS, NLS-3xV5, and NLS-3xc-myc flanked by *attB2R*-*attB3* sites were designed in Vector NTI^®^ (Invitrogen) and ordered from GenScript as clones in the pUC57 vector. These tags were introduced into pDONR P2R-P3 through a BP reaction. The resulting entry vectors were transformed into *E. coli* and sequence verified.

Total yeast protein extracts were obtained as described [41] and concentration quantified using the Bio-Rad Protein Assay (Bio-Rad). Samples were combined with 5x Laemmli loading buffer and denatured for 10 min at 95°C. Subsequently, 30 μg total protein was loaded on a 4–15% Mini-PROTEAN^®^ TGX™ Precast Gel (Bio-Rad) and transferred to a PVDF membrane using the Trans-Blot Turbo transfer system (Bio-Rad). Detection was performed using the following primary antibodies: anti-FLAG (Sigma), anti-c-myc-HRP (Invitrogen), anti-HA (Roche), and anti-V5 (Sigma).

### Yeast two- and three-hybrid

The primers were designed to clone the ORF corresponding to TPL-N with and without STOP codon (Additional file 1) [38]. The entry clones pEN-L4-*GPD*-R1, pEN-R2-*NLS-3xFLAG-6xHis*-L3, and pEN-L1-*NINJA*-L2 were recombined by MultiSite Gateway^TM^ LR reaction with pMG426 as destination vector. Construction of the pGADT7- and pGBKT7-clones, and the Y2H and Y3H were carried out as described [33] except that transformed yeast cells (strain PJ69-4a) were selected on SD-Ura-Trp-Leu.

## Competing interests

The authors declare that they have no competing interests.

## Authors’ contributions

AND, TM and RDC carried out experiments. LP and AG designed experiments. AND and LP wrote the manuscript. All authors read, edited and approved the manuscript.

## Supplementary Material

Additional file 1 Primers used and their sequence. Click here for file
