# Supplementary material for: A MultiSite GatewayTM vector set for the functional analysis of genes in the model Saccharomyces cerevisiae
Source: BMC Mol Biol. 2012 Sep 20;13:30. doi: 10.1186/1471-2199-13-30 (PMC3519679; doi:10.1186/1471-2199-13-30)
Supplement: Additional file 1 — Primers used and their sequence. [file 1471-2199-13-30-S1.doc]

# Additional files

### Additional file 1. Supplementary Information

## Table S1. Primers used and their sequence.

| Primer | Sequence |
| --- | --- |
| XhoI-attB4 | GCAGTACGGAGCTCCAACTTTGTATAGAAAAGTTG |
| SacI-attB3 | GATAGTGTCTCGAGCAACTATGTATAATAAAGTTG |
| pGPD-Fw | ATAGAAAAGTTGTTGGATCCGTCGAAACTAAG |
| pGPD-Rv | TGTACAAACTTGTACTGCCATTTCAAAGAATACG |
| pGAL1-Fw | AGAAAAGTTGAACAACTTCTTTTC |
| pGAL1-Rv | GTACAAACTTGTAGTTGATTGTAT |
| pADH1-Fw | ATAGAAAAGTTGTTACGGATTAGAAGCCGCC |
| pADH1-Rv | TGTACAAACTTGTGGTTTTTTCTCCTTGACGTTA |
| M13-Fw | GTAAAACGACGGCCAGT |
| M13-Rv | CCAGGAAACAGCTATGACCAT |
| TPL-N-Fw | GGGGACAAGTTTGTACAAAAAAGCAGGCTCCATGTCTTCTCTTAGTAGAGAG |
| TPL-N-Rv | GGGGACCACTTTGTACAAGAAAGCTGGGTCTCMATTTTTACAAAGCTGGTGTTG |
